# Ovarian endometrioma infiltrating neutrophils orchestrate immunosuppressive microenvironment

**DOI:** 10.1186/s13048-020-00642-7

**Published:** 2020-04-25

**Authors:** Hua Xu, Jing Zhao, Jiaqi Lu, Xiaoxi Sun

**Affiliations:** 1 Obstetrics and Gynecology Hospital, Fudan University, Shanghai, 200011 China; 2 Shanghai Ji Ai Genetics and IVF Institute, Obstetrics and Gynecology Hospital, Fudan University, Shanghai, 200011 China; 3 Department of Gynecology, Second People’s Hospital in Kashgar, Xinjiang Uyhur Autonomous Region. No.1, Health Road, Kashgar, 844000 Xinjiang China; 4 Key laboratory of Female Reproductive endocrine Related Diseases, Obstetrics and Gynecology Hospital, Fudan University, Shanghai, 200011 China

**Keywords:** Ovarian endometrioma, Neutrophils, PD-L1, Lymphocytes, Immunosuppression

## Abstract

**Background:**

Ovarian endometrioma (EM) lesions not only have overwhelmed the amount of infiltrating immune cells but also display immunosuppressive phenotype. The close relationship between neutrophils and the pathogenesis of endometriosis has been demonstrated. The present study aims to elucidate whether or not neutrophils are involved in the regulation of immunosuppressive microenvironment in ovarian endometrioma.

**Methods:**

Immunochemistry (IHC) and flow cytometry analysis (FACS) were conducted to measure CD66b expression in ovarian endometrioma samples from EM patients. The correlation between percentage of CD66b and PD1 + CD8+, TIM3 + CD8+, CTLA4 + CD8+, IFN-γ + CD8+ of CD45+ cells were analyzed. Neutrophil survival and PD-L1 expression were determined under the stimulations of ovarian endometrioma conditional supernatants (OECS). Finally, CD8+ T cell’s proliferation and IFN-γ expression were detected under co-cultured with OECS cultured neutrophils stimulated with the α-CD3/α-CD28 antibody.

**Results:**

IHC and FACS results revealed correlation between the counts of neutrophils and the severity of ovarian endometrioma. The percentage of CD66b + cells was positively correlated with PD1 + CD8+, TIM3 + CD8+ and CTLA4 + CD8+ of CD45+ cells in ovarian endometrioma. OECS promoted neutrophils’ survival and enhanced PD-L1 expression. OECS cultured neutrophils inhibited proliferation and activity of autologous T cells.

**Conclusions:**

Neutrophils play a crucial role in the progression of ovarian endometrioma by orchestrated the immunosuppressive microenvironment under the PD-1/PD-L1 axis.

## Introduction

Endometriosis (EM) is defined as a chronic inflammatory disease characterized by ectopic presence of endometrial glands and stroma predominantly in the pelvic compartment, and is a common disorder affects women of reproductive period with pelvic pain and reduced fertility [1]. Three types of EM have been characterized: peritoneal superficial endometriosis (SUP), ovarian endometrioma (OMA), and deeply infiltrating endometriosis(DIE) [2, 3]. The involvement of various immune cells such as T and B lymphocytes, natural killer cells, macrophages, and dentritic cells have been demonstrated in peritoneal inflammation and endometriosis lesion establishment [4, 5]. Endometriosis lesions not only have overwhelmed amount of infiltrating immune cells, but these cells often exhibit immunosuppressive phenotype, suggesting that defects of immune surveillance generated by suboptimal immune response that lead to the initiation and development of endometriosis [6].

Recently, the close relationship between neutrophils and the pathogenesis of endometriosis has been demonstrated. For instance, accumulation of neutrophils has been found in the peritoneal fluid of ovarian endometriosis [7, 8]. Neutrophil extracellular traps (NETs) produced by activated neutrophils are present in the peritoneal fluid of endometriosis patients [9]. Neutrophil infiltrating the human endometrium express VEGF and regulate cyclical endometrial vascular proliferation [10]. Endometriosis is associated with increased percentage of peritoneal neutrophils and that the levels of human neutrophil peptides (HNP 1–3) strongly correlated with the percentage of neutrophils [8]. Depletion neutrophils significantly reduced the severity of endometriotic lesions [11]. However, the role of neutrophil in the immunosuppressive microenvironment of ovarian endometrioma remains unknown.

Neutrophils are highly plasticity implying that these cells may differentiate into discrete subsets under pathological conditions [12]. For instance, neutrophils display not only protumor but also anti-tumor properties depending on tumor growth factor-β (TGF-β) within the tumor microenvironment [13]. Neutrophils adopted anti-inflammatory phenotype during *Mycobacterium tuberculosis* infection [14]. Furthermore, neutrophils contribute to orientation of adaptive immune responses by suppression of T cell proliferation and activity under the control of interleukin-10 (IL-10), arginase 1, and reactive oxygen species [15]. In the gastric cancer, tumor activated neutrophils foster immune suppression may be a result of PD1/PD-L1 axis [16]. Notably, the phenotype and function relevance of neutrophils in the immunosuppressive microenvironment of ovarian endometriosis remain unknown.

Herein, we found the association between the severity of endometriosis and the counts of infiltrating neutrophils. Moreover, we demonstrated that ovarian endometrioma prolonged neutrophil lifespan and induced programmed death-ligand 1 (PD-L1) expression on neutrophils. In turn, these neutrophils suppress T cell’s proliferation and activation, indicating PD-L1 expression neutrophils may contribute to the suppression of adaptive immune response during the progression of ovarian endometrioma.

## Materials and methods

### Ovarian endometrioma tissue and peripheral blood collection

Endometriotic tissue specimens were obtained from 56 women with ovarian endometrioma who underwent laparoscopic surgery at the Department of Obstetrics and Gynecology, Obstetrics and Gynecology Hospital, Fudan University, from March 2016 to Jun2016. Cohort 1 (*n* = 35) were subjected to immunohistochemical analysis. Cohort 2 (*n* = 21) were subjected to flow cytometry analysis. Their major clinical and surgical features are presented in Table 1. The clinical characteristics are balance between two cohorts. All ovarian endometrioma subjects were histologically proven endometrioma. During surgery, endometriosis was staged and scored (total, implant, and adhesion scores) according to the revised American Fertility Society (rAFS) classification [17]. Women with isolated ovarian endometrioma corresponded to women with ovarian endometrioma associated peritoneal endometriosis with adhesions, exclusive of deeply infiltrative endometriosis [18]. Furthermore, women with infectious diseases such as hepatitis B or C virus, or human immunodeficiency virus and women with malignancies or autoimmune diseases were excluded in this study. None of the included women were pregnant.

**Table 1:** Clinical characteristics of 56 cases with ovarian endometriosis

Characteristic	Cohort 1(*n* = 35)	Cohort 2 (*n* = 21)	*P* value
Age (yr)	31 (23–46)	32 (23–44)	0.461
Name of operation			0.983
Laparoscopic right ovarian cystectomy	9 (25.71%)	5 (23.81%)	
Laparoscopic left ovarian cystectomy	11 (31.43%)	7 (33.33%)	
Laparoscopic cystectomy of both ovaries	15 (42.86%)	9 (42.86%)	
Endometriosis score by rASRM criteria	39 (22–132)	41 (28–130)	0.174
The largest diameter of endometrioma (cm)	6 (3–10)	7 (1.5–11)	0.182
Dysmenorrhea	21 (60%)	10 (47.62%)	0.415

Values are presented as mean (range) or number (%)

*rASRM* revised American Society for Reproductive Medicine

Whole blood collected from 10 health adult female donors (aged 20–50 years) was used to isolate neutrophils and peripheral blood mononuclear cells (PBMCs).Neutrophils and PBMC prepared using PolymorphPrep (Axis-Shield) and LymphoPrep (Axis-Shield) following the manufacturer’s protocol. This study was approved by the Ethics Committee of the Affiliated Obstetrics and Gynecology Hospital of Fudan University. Written informed consent was obtained from each participant in accordance with the approved guidelines.

### Immunohistochemistry

Paraffin sections (5 μm) of ovarian endometrioma tissue were deparaffinized in xylene, rehydrated in graded ethanol, and subjected to antigen retrieval using sodium citrate buffer (10 mM, pH 6.0). The sections were blocked with H_2_O_2_ and blocking buffer (2.5% bovine serum albumin (BSA) in phosphate buffer saline (PBS). The sections were then incubated with mouse anti-human CD66b (1:600, Biolegend). Horseradish peroxidase conjugated secondary antibodies (Vector Laboratories) were applied for 30 min and visualized with 3′3-diaminobenzidine (Vector Laboratories). Staining with isotype antibody was used as negative control. Images were taken on the Nikon Eclipse 80i microscope (Nikon, Japan). The neutrophil was determined as number of cells/high power field (HPF) using an algorithm developed for National Institutes of Health (NIH) software ImageJ.

### Preparation of OECS and supernatant-conditioned neutrophils

Ovarian endometrioma tissue culture supernatants (OECS) were prepared by plating ovarian endometrioma tissues in 1 mL RPMI-1640 medium for 24 h. The supernatant was then centrifuged and harvested. Four respect ovarian endometrioma specimens were prepared for OECS. To generate supernatant-conditioned neutrophils, neutrophils were cultured with 50% OECS for 12 h, then washed with RPMI-1640 medium for three times. Neutrophils cultured with RPMI-1640 medium were used as controls.

### Neutrophils survival assay

Neutrophils from healthy donors were stimulated with30% or 60% OECS for 16 h, and then were harvested. Neutrophils survival was quantified using Annexin V Apoptosis Detection Kit (BD biosciences) according to the manufacturer’s instructions.

### Measurement of CD8+ T cells proliferation with CFSE

Measurement of CD8^+^ T cells proliferation with5-(and 6)-Carboxyfluorescein diacetate succinimidyl ester (CFSE) was applied as previously described [19]. PBMC were resuspended at 1 × 10^7^ cells/mL in PBS containing 5 μM CFSE (Biolegend). After 20 min incubation at room temperature, cells were washed twice with culture medium. Then the PBMC were stimulated with anti-CD3 Ab (1 μg/mL), anti-CD28 Ab (1 μg/mL) and IL-2 (100 U/mL) in the presence or absence of OECS conditioned neutrophils for 3 days. Cells were then stained with CD8 mAb, and CFSE positive CD8^+^ T cells were finally analyzed by a flow cytometer (Beckman CytoFLEX) using CytExpert Software.2.7.

### Analysis of intracellular IFN-γproduction by flow cytometry

PBMC (2 × 10^6^ cells/mL) were stimulated with immobilized anti-CD3/anti-CD28 (each at 1 μg/mL) and IL-2 (100 U/mL) in the absence or presence of OECS conditioned neutrophils. After 24 h, 1 × protein transport inhibitor cocktail (Invitrogen) was added for the last 6 h. Following stimulation, cells were harvested, washed in PBS and incubated for 30 min at 4 °C in the darkness with the anti-CD8 mAb (Biolegend). Cells were then washed once in PBS containing 2% v/v FCS prior to fixation and permeabilization with 500 μL Cytofix/Cytoperm solution (Becton Dickinson) for 10 min at room temperature in darkness. Cells were washed once again and incubated with blocking buffer for 20 min at 4 °C in the darkness to prevent unspecific binding of the anti-cytokine monoclonal antibody. After another washing step, 5 μL anti-IFN-γ-FITC (Biolegend) were added and cells were incubated for 30 min at 4 °C in the darkness followed by a final washing step prior to measurement. Measurement was done using flow cytometer (Beckman CytoFLEX) using CytExpert Software.2.7.

### Statistical analysis

Student’s t tests were used to compare differences in the means of the continuous variable between the groups of patients with two cohorts of endometriosis. The relationship between the endometriosis score and each of parameters were evaluated using the nonparametric Spearman’s rank correlation test. All statistical analyses were performed using Prism (Graphpad). A *P*-value < 0.05 was considered statistically significant.

## Results

### Patient characteristics

Fifty-six ovarian endometrioma affected women were recruited for this study. Their major clinical and surgical features are presented in Table 1.Ovarian endometrioma patients were distributed among rAFS stages III and IV as follows: 24 (42.9%) and 32 (57.1%), respectively. Mean total, implants, and adhesion rAFS scores were 56.1 ± 31.4, 27.3 ± 8.7, 28.6 ± 27.4, respectively.

### Neutrophils accumulated in the ovarian endometrioma

**Fig. 1:**
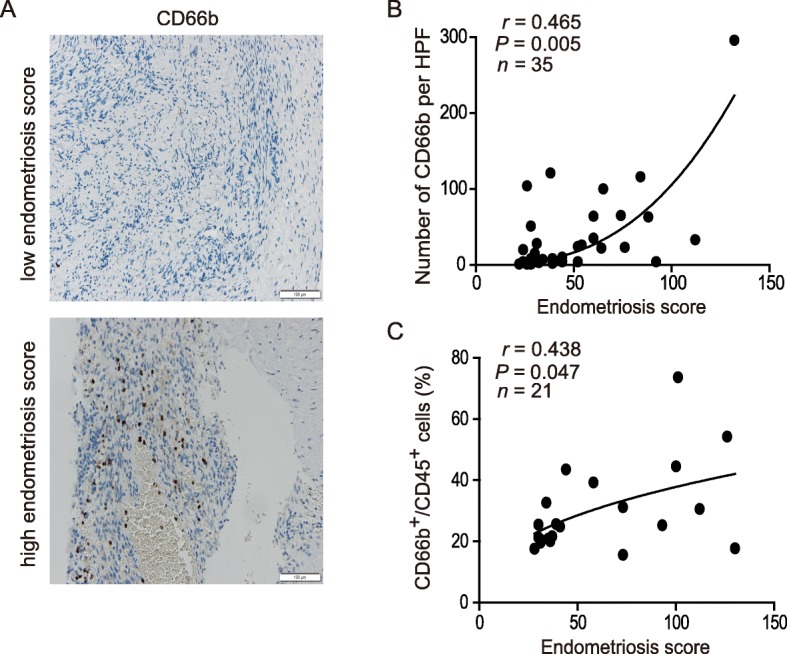
Ovarian endometriosis infiltrating neutrophils were correlated with endometriosis scores. **a** Representative image showing neutrophils infiltrating in ovarian endometriosis specimens. Bar = 100 μM. **b** The correlation between the endometriosis scores and the number of CD66b per high power field (HPF) in ovarian endometriosis specimens detected by IHC (*n* = 35). **c** The correlation between the endometriosis scores and the percentage of CD66b^+^ of CD45^+^ cells in ovarian endometriosis specimens detected by FACS (*n* = 21)

We detected neutrophil infiltration in ovarian endometrioma specimens with CD66b staining in immunohistochemistry. The results showed that the amount of infiltrating neutrophils was positively correlated with the severity scores of patients with ovarian endometrioma (*r* = 0.465, *P* = 0.005) (Fig. 1a and b). Consistently, the association between the severity of ovarian endometrioma and the percentage of CD66b^+^ of CD45^+^ cells were confirmed (*r* = 0.438, *P* = 0.047) (Fig. 1c).

### Neutrophils infiltrating ovarian endometrioma were defined with immunosuppressive phenotype

**Fig. 2:**
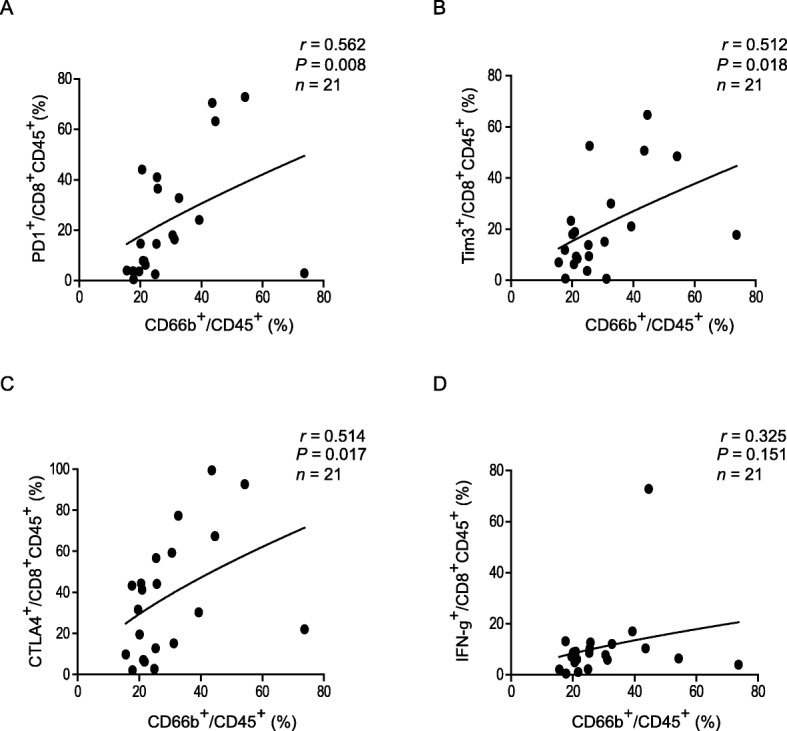
Ovarian endometriosis infiltrating neutrophils were correlated with the number of immune checkpoints positive CD8 T cells. **a**-**d** The correlation between the percentage of CD66b^+^ of CD45^+^ cells and the percentage of PD1^+^ of CD8^+^CD45^+^ lymphocytes (A), the percentage of Tim3^+^of CD8^+^CD45^+^ lymphocytes (**b**), the percentage of CTLA4^+^of CD8^+^CD45^+^ lymphocytes (C), the percentage of IFN-γ^+^ of CD8^+^CD45^+^ lymphocytes (**d**)

Next, significant positive correlations were found between the levels of CD66b^+^ and PD1^+^, Tim3^+^, and CTLA4^+^ expression on CD8^+^ T cells (Fig. 2a-c). However, there was no correlation between the levels of CD66b^+^ and IFN-γ^+^ expression on CD8^+^ T cells (Fig. 2d).

### Ovarian endometrioma environments contribute to neutrophil survival and maintain neutrophil activated immunosuppressive phenotype

**Fig. 3:**
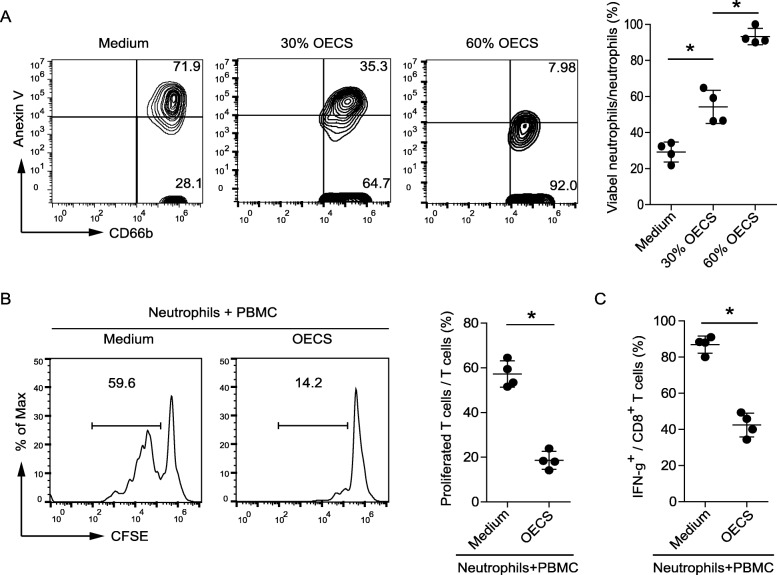
Ovarian endometriosis environment sustains neutrophil survival, and ovarian endometriosis cultural supernatants conditioned neutrophils suppress T cell immunity. **a** Dot plots and statistics analysis of annexin V^—^viable non-apoptotic neutrophils exposed to 30 and 60% ovarian endometriosis cultural supernatants (OECS) from ovarian endometriosis patients for 16 h. **b** Human PBMC were labeled with CFSE and proliferation was induced by anti-CD3/anti-CD28 mAb (1 μg/mL each) in the presence of IL2 (100 U/mL). The degree of proliferation was assessed by analyzing the reduction of CFSE-label after cell division in CD8^+^ lymphocytes by flow cytometry. One representative out of four experiments is shown in the left. Statistical analysis of thepercentage of CD8+ T cell proliferation as shown in the right. (*n* = 4, * *P* < 0.05). **c** Human PBMC were stimulated for 24 h by anti-CD3/anti-CD28 mAb (1 μg/mL each) and 1 × protein transport inhibitor cocktail (Invitrogen) added for the last 6 h. Cells were then stained with CD8 mAb, and intracellular IFN-γ production in CD8^+^ T cells was analyzed by flow cytometry. Statistical analysis of the percentage of IFN-γ^+^ of CD8^+^ T cells was shown

We hypothesized that ovarian endometrioma environment sustained the survival of neutrophils. To test this hypothesis, we assessed the survival of neutrophils after exposure to OECS, and the results showed that neutrophils exposed to OECS exhibited a delayed onset of apoptosis in a dose-dependent manner when compared to those exposed to medium by annexin V (Fig. 3a). Given the immunosuppressive microenvironment in the ovarian endometrioma, we hypothesized that the endometrioma microenvironment itself might play an important role in this process. PBMC were co-cultured with OECS-conditioned neutrophils. OECS-conditioned neutrophils significantly suppressed T cell proliferation and less IFN-γ production (Fig. 3b, C).

### PD-L1 expression in neutrophils are found in ovarian endometrioma

**Fig. 4:**
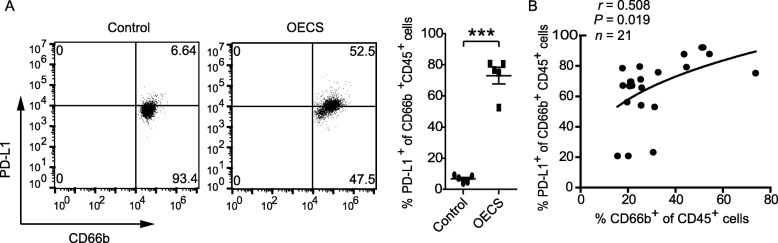
Ovarian endometriosis environment sustains PD-L1 expression on neutrophils. **a** Dot plots and statistics analysis of PD-L1^+^ expression on neutrophils exposed to 60% ovarian endometriosis cultural supernatants (OECS) from ovarian endometriosis patients for 12 h. (*n* = 5, ***P < 0.001). **b** The correlation between the percentage of CD66b^+^ of CD45^+^ cells and the percentage of PD-L1^+^ of CD66b^+^CD45^+^cells

Meanwhile, we also hypothesized that OECS environments contribute to the immunosuppressive phenotype of neutrophils. Consistent with our hypothesis, compared with medium-conditioned neutrophils, OECS significantly up-regulated PD-L1 expression (Fig. 4a). Consistently PD-L1 expression levels were positively correlated with the percentage of CD66b^+^cells in ovarian endometrioma specimens (Fig. 4b).

## Discussion

Endometriosis is a chronic inflammatory disease driven by dysregulated immune system, which facilitates endometrial cells implantation, proliferation, and promotes molecular abnormalities within the eutopic endometrium [20]. Peritoneal neutrophils and macrophage-driven inflammatory networks enhance endometriotic cell survival, cell growth, cell invasion, and angiogenesis [11, 21]. Suppression of cytotoxicity of NK cells and imbalance of T cell subsets has limited capability to eliminate endometrial cells in the peritoneal cavity [22, 23]. In contrast, studies that focus on endometriosis lesion infiltrating immune cells are very limited. Here, we found statistically significant correlations between the ovarian endometrioma infiltrating neutrophils and the severity of endometriosis. Ovarian endometrioma microenvironment induced PD-L1 expression in neutrophils, which acquired the ability to suppress CD8 T cell proliferation and activation.

Neutrophils are the first line to defense against microbial infections and play a pivotal role in inflammatory disease [24]. In the peripheral fluid of endometriosis, increased amounts of neutrophils has been observed, especially in the advanced stages [8, 25]. In the blood of endometriosis, increased neutrophil-to-lymphocyte ratio has been found and proposed as a potential measure of disease severity [22, 26]. Moreover, a substantial infiltration of neutrophils into the peritoneal cavity occurred during early initiation of uterine tissue transfer mouse model of endometriosis [27]. Early depletion of neutrophils by anti-granulocyte receptor (Gr-1) antibody relieved the formation of endometriotic lesion, which suggests that neutrophils play an important role in the initiation and progression of endometriosis. In the present study, we found that amounts of lesion infiltrating neutrophils were correlated with the severity of the ovarian endometrioma.

CXC motif ligand 8 (CXCL8/IL8), the most potent neutrophil chemoattractant, contributes to recruit neutrophils by binding to the receptors CXCR1 and CXCR2. Other chemokines such as CXCL1, CXCL2, CXCL3, and G-CSF can also attract neutrophils [28]. Consistently, patients with endometriosis displayed a significant higher production of CXCL1, CXCL2, and CXCL8 in the peritoneal fluid as compared to those from controls [29–31]. Secretion of CXCL8 is significantly higher in the eutopic endometrial stromal cells from women with endometriosis than that in the control [32]. The elevated concentration of neutrophil chemoattractants may be responsible for the recruitment of neutrophils in the peritoneal fluid and endometriosis lesion.

Neutrophils not only promote endometriosis growth by secreting a variety of cytokines and chemokines, but also enhance endometriosis angiogenesis, migration and invasion by providing enzymes that deregulate the extracellular matrix (ECM). Neutrophils are able to release numerous mediators, including pro-inflammatory (e.g., IL-1α/β, IL-6, IL-8, IL-9, IL-16), angiogenic (e.g., vascular endothelial growth factor (VEGF) and anti-inflammatory mediators (e.g. tumor growth factor (TGF-β), IL-4, IL-10) [15].Within the surrounding peritoneal fluid, numerous cytokines (e.g., TNF-α, IL-10, and TGF-β), chemokines (e.g., IL-8), angiogenic growth factors (e.g., VEGF) are present [33]. Neutrophils in the peritoneal fluid of endometriosis increase the production of VEGF in the presence of IL-6, TNF-α, LPS, and estrogen [34]. Matrix metalloproteinase (MMP9) expression increased in the endometrium from women with endometriosis [35]. Furthermore, higher neutrophil extracellular traps (NETs) levels in the peritoneal fluid of patients with endometriosis has been found, which seem to be implicated in its pathogenesis [9]. Neutrophils also contribute to immune suppression to enhance endometriosis growth. This together with the fact that IFN-γ stimulated PD-L1 expression in neutrophils and acquire the capacity to suppress T cell proliferation [36]. Although neutrophils have already been described in patients with endometriosis, the alterations of functions of neutrophils in the endometriosis microenvironment remain to be elucidated. Here, we found that increased PD-L1 expression in neutrophils in the presence of ovarian endometrioma supernatants, which inhibit CD8 T cell proliferation and activation.

The primary limitation of our study included only women who underwent surgery for ovarian endometrioma and were diagnosed with moderate to severe endometriosis. There is lack of adjacent healthy ovarian tissues applied for preparation of culture supernatants. In summary, this study demonstrates that neutrophils are correlated with the severity of ovarian endometrioma and inhibit CD8 T cell immunity under PD-L1 dependent manner, although factors that mediate neutrophil PD-L1 expression required further investigation. These findings add to our current understanding of disease development and inform new strategies for the prevention and management of ovarian endometrioma.

## Conclusions

In conclusion, our results indicated that the amount of neutrophils was associated with the severity of ovarian endometriosis. The ovarian endometriosis associated neutrophils inhibits the activities of CD8+ T cells in vitro, and may play a role in the development of immunosuppressive microenvironment by PD-L1 expression in vivo. Moreover, these conclusions should be validated by more stringent controls.

## Data Availability

All publicly data generated or analyzed during this study are included in this published article. The data used to support the findings of this study are available from the corresponding author upon request.

## Abbreviations

BSA: Bovine serum albumin
CFSE: 5-(and 6)-carboxyfluorescein diacetate succinimidyl ester
CXCL8: cxc motif ligand 8
DIE: Deeply infiltrating endometriosis
EM: Endometrioma
FACS: Flow cytometry analysis
HNP: Human neutrophil peptides
Gr: Granulocyte receptor
HPF: High power field
IHC: Immunochemistry
IL: Interleukin
MMP: Matrix metalloproteinase
NETs: Neutrophil extracellular traps
NIH: National Institutes of Health
OECS: Ovarian endometrioma conditional supernatants
OMA: Ovarian endometrioma
PBMC: Peripheral blood mononuclear cells
PBS: Phosphate buffer saline
PD-L1: Programmed death ligand 1
Rafs: Revised American Fertility Society
SUP: Peritoneal superficial endometriosis
TGF-β: Tumor growth factor-β
VEGF: Vascular endothelial growth factor
